# Topography of the respiratory tract bacterial microbiota in cattle

**DOI:** 10.1186/s40168-020-00869-y

**Published:** 2020-06-10

**Authors:** Christopher McMullen, Trevor W. Alexander, Renaud Léguillette, Matthew Workentine, Edouard Timsit

**Affiliations:** 1 Faculty of Veterinary Medicine, University of Calgary, Calgary, Alberta Canada; 2 Lethbridge Research and Development Center, Agriculture and Agri-Food Canada, Lethbridge, Alberta Canada; 3 Simpson Ranch Chair in Beef Cattle Health and Wellness, University of Calgary, Calgary, Alberta Canada; 4 Ceva Santé Animale, 10 Avenue de la Ballastière, 33500 Libourne, France

**Keywords:** Microbiome, Bovine respiratory disease, Natural cattle, Antibiotic free

## Abstract

**Background:**

Bacterial bronchopneumonia (BP) is the leading cause of morbidity and mortality in cattle. The nasopharynx is generally accepted as the primary source of pathogenic bacteria that cause BP. However, it has recently been shown in humans that the oropharynx may act as the primary reservoir for pathogens that reach the lung. The objective was therefore to describe the bacterial microbiota present along the entire cattle respiratory tract to determine which upper respiratory tract (URT) niches may contribute the most to the composition of the lung microbiota.

**Methods:**

Seventeen upper and lower respiratory tract locations were sampled from 15 healthy feedlot steer calves. Samples were collected using a combination of swabs, protected specimen brushes, and saline washes. DNA was extracted from each sample and the 16S rRNA gene (V3-V4) was sequenced. Community composition, alpha-diversity, and beta-diversity were compared among sampling locations.

**Results:**

Microbiota composition differed across sampling locations, with physiologically and anatomically distinct locations showing different relative abundances of 1137 observed sequence variants (SVs). An analysis of similarities showed that the lung was more similar to the nasopharynx (R-statistic = 0.091) than it was to the oropharynx (R-statistic = 0.709) or any other URT sampling location. Five distinct metacommunities were identified across all samples after clustering at the genus level using Dirichlet multinomial mixtures. This included a metacommunity found primarily in the lung and nasopharynx that was dominated by *Mycoplasma*. Further clustering at the SV level showed a shared metacommunity between the lung and nasopharynx that was dominated by *Mycoplasma dispar*. Other metacommunities found in the nostrils, tonsils, and oral microbiotas were dominated by *Moraxella*, *Fusobacterium*, and *Streptococcus*, respectively.

**Conclusions:**

The nasopharyngeal bacterial microbiota is most similar to the lung bacterial microbiota in healthy cattle and therefore may serve as the primary source of bacteria to the lung. This finding indicates that the nasopharynx is likely the most important location that should be targeted when doing bovine respiratory microbiota research.

Video abstract.

## Background

Bacterial bronchopneumonia (BP), often referred to as bovine respiratory disease, persists as one of the most significant diseases facing the cattle industry worldwide [1, 2]. The bacterial pathogens most commonly associated with BP include *Mannheimia haemolytica*, *Pasteurella multocida*, *Histophilus somni*, and *Mycoplasma bovis* [2].

It is generally accepted that the nasopharynx is the primary source of the pathogenic bacteria that cause BP [3, 4]. Indeed, the nasopharynx has been implicated as having a causative role in the pathogenesis of the disease [5]. Despite this, it has never been definitively shown that the nasopharynx acts as the primary reservoir for bacterial pathogens that cause lung infection.

Microbiotas within different niches of the upper respiratory tract (URT) could potentially contribute more to the lung microbiota than the nasopharynx. Recent research in humans suggests that the oropharynx may be the primary source for bacterial respiratory pathogens [6, 7]. Concomitant analysis of the upper and lower respiratory tract microbiotas revealed that the nasal microbiota contributed little to the composition of the lung microbiota in healthy humans [6, 7]. Comparatively, the mouth and oropharynx contributed the most to the composition of the lung microbiota [6, 7]. The tonsils may also act as a possible source of bacteria translocating to the lungs in cattle. Culture-based results in cattle have shown that the tonsils can harbor BP pathogens [8].

Elucidating the contributions of different URT niche bacterial microbiotas to bacteria in the lung is important to better understand the pathogenesis of BP and develop mitigation technologies that target BP-associated pathogens. Most studies on BP (diagnostics, antimicrobial resistance, pathogenesis) are based on samples collected from the nasopharynx (i.e., deep nasal swabs) [3, 4, 9, 10]. Furthermore, recently tested control strategies for BP focused only on the nasopharyngeal microbiota (e.g., nasal instillation of nitric oxide or probiotics) [11, 12]. Describing the microbial composition of the URT is crucial for the research and development of new diagnostic and control strategies for BP so that we focus our efforts on only the most relevant bacterial communities (e.g., oropharyngeal and/or tonsillar microbiotas). Therefore, this study was designed with the objective to describe the bacterial microbiotas present along the entire cattle respiratory tract to determine which URT niches may contribute the most to the composition of the lung microbiota.

## Results

### Health data

A total of 18 steers were sampled, but only 15 healthy steers (arrival bodyweight = 342 ± 33 kg) remained in the study after three were excluded due to increased serum haptoglobin levels. These steers arrived at the feedlot an average of 12 ± 2 days before enrollment to the study. Average rectal temperature at enrollment was 39.5 ± 0.4 °C. None of the steers had lung consolidations or pleural effusion detected at thoracic ultrasonography.

### Baseline sequencing data

A total of 19,307,004 reads were obtained across all samples from two sequencing runs (Run 1 = 10,574,638; Run 2 = 8,732,366) with an average Phred quality score of 33.9 (Run 1 = 33.9; Run 2 = 33.8) prior to upstream processing. Two individual samples (one from the primary bronchus of the left caudal lobe and one from the secondary bronchi of the left caudal lobe) from two different steers were removed from the study for having insufficient sequencing depth (< 500 sequencing reads/sample). After processing with DADA2 and removing the two aforementioned samples, a total of 8,270,814 reads remained across all samples (Run 1 = 4,273,884; Run 2 = 3,996,930), with an average of 32,691 reads per sample (range = 1104-66,288). From these sequences, 6210 unique sequence variants (SVs) were identified across all samples. After removal of all SVs that did not belong to the kingdom Bacteria, a total of 6139 SVs remained. Furthermore, after 1% prevalence filtering 1137 SVs remained across all samples.

Along with the various study samples, 30 negative control and 2 positive control samples were sent for targeted amplicon sequencing. The sequencing results for each negative control were assessed individually, and it was determined that, based on the composition of the reads and the extremely low numbers of sequences returned for each sample, there was insignificant contamination of the study samples due to improper sample collection and storage techniques or the DNA extraction and targeted amplicon sequencing processes (Additional file 1). Therefore, there was no need to adjust the study sample DNA sequencing results for possible contaminants. Both positive control samples were assessed individually, and it was determined that, based on representation of all 10 expected mock community bacteria, the DNA extraction and targeted amplicon sequencing processes were valid and reliable (Additional file 1). All sequencing data for the study samples, negative controls, and positive controls were deposited to the National Center for Biotechnology Information Sequence Read Archive under accession number PRJNA596300.

### Characterization of the respiratory tract bacterial microbiotas

The most prominent phyla across all sampling locations were *Proteobacteria* (27.11%), *Tenericutes* (22.38%), *Firmicutes* (21.36%), *Actinobacteria* (14.00%), *Fusobacteria* (9.81%), and *Bacteroidetes* (5.05%) (Additional file 2); however, the order of phyla by mean relative abundance differed by sampling location (Additional file 3). *Proteobacteria* was most abundant in the nostrils, nasopharynx, and oropharynx; *Firmicutes* was most abundant on the floor of the mouth and hard palate, and *Fusobacteria* was most abundant in the tonsils. In general, *Tenericutes* was most abundant in sampling locations from the distal trachea down into the lung, except for the secondary bronchi of the left and right caudal lobes where *Actinobacteria* was highest.

**Fig. 1:**
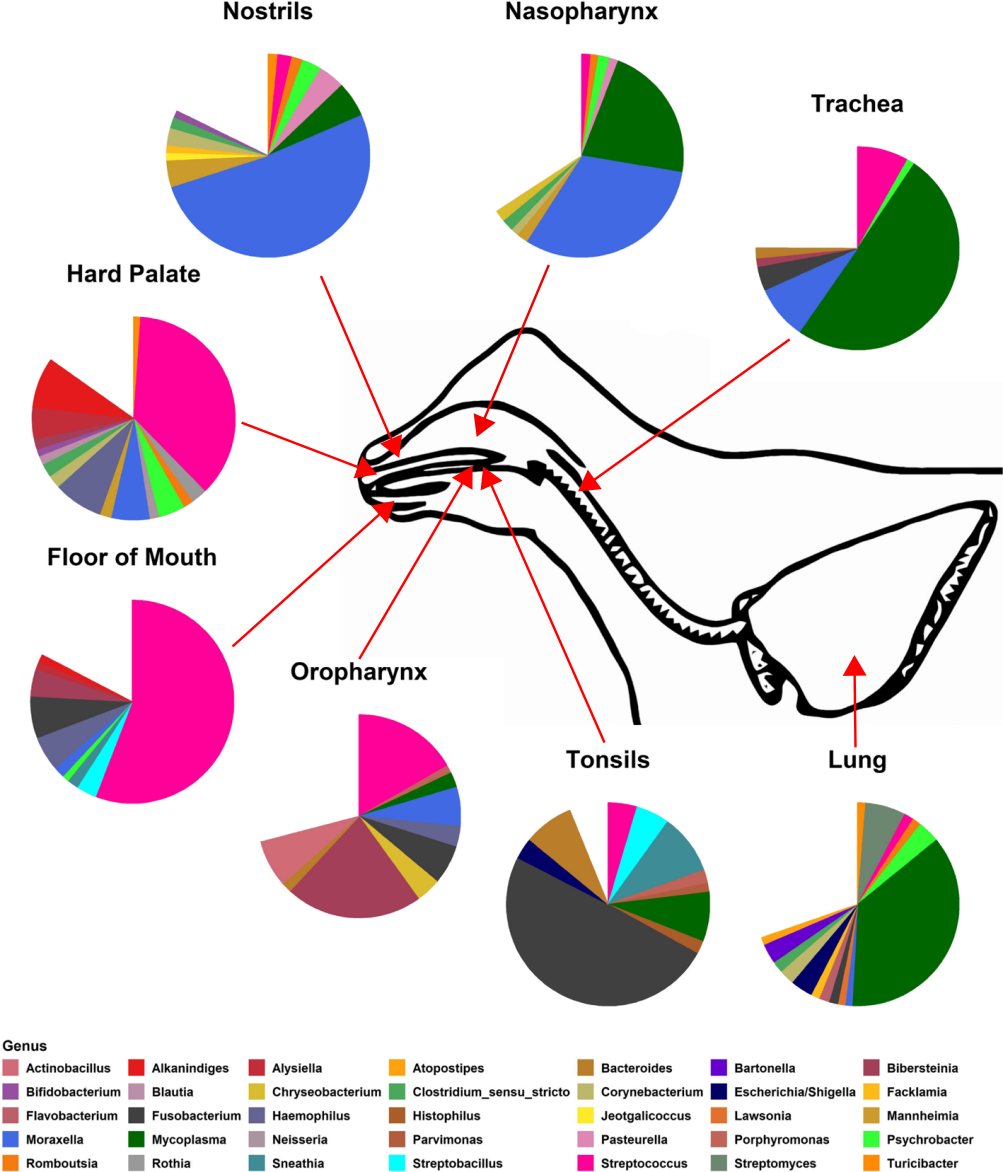
Mean relative abundance of bacteria present at ≥ 1% abundance at the genus level of different upper and lower respiratory sampling location metagroups for 15 healthy beef steer calves

The five most prominent genera across all sampling locations were *Mycoplasma* (22.28 %), *Moraxella* (11.65%), *Streptococcus* (8.56%), *Fusobacterium* (7.48%), and *Streptomyces* (2.63%) (Additional file 2); however, the order of genera by mean relative abundance differed by sampling location (Additional file 4). *Moraxella* was most abundant in the nostrils and nasopharynx, whereas *Streptococcus* was most abundant on the floor of the mouth and hard palate and *Bibersteinia* was most abundant in the oropharynx (Fig. 1). *Fusobacterium* was most abundant in the tonsils and *Mycoplasma* was most abundant in the trachea and lung.

The five most prominent species across all sampling locations were *Mycoplasma dispar* (16.02 %), *Fusobacterium necrophorum* (7.26%), a *Streptococcus* SV ambiguously identified to > 5 species (2.63%), an *Escherichia/Shigella* SV ambiguously identified to > 5 species (1.75%), and *Moraxella bovoculi* (1.49%) (Additional file 2); however, the order of species by mean relative abundance differed by sampling location (Additional file 5).

### Comparison of bacterial microbiota structure within and between sampling locations

**Fig. 2:**
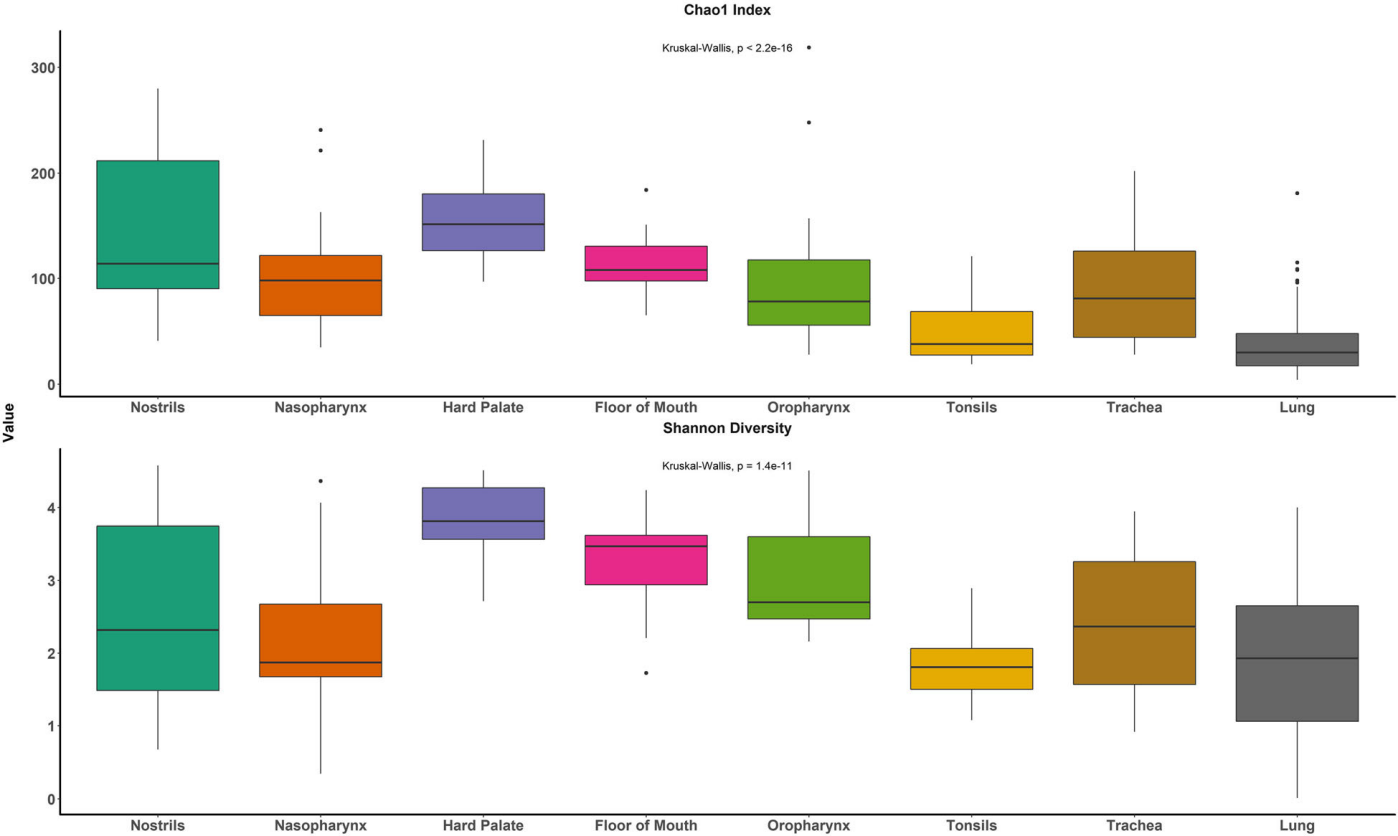
Species richness (Chao1 index) and diversity (Shannon diversity index) of different upper and lower respiratory sampling location metagroups for 15 healthy beef steer calves

Species richness (Chao1 index) mainly decreased from the proximal URT locations to the lower respiratory tract locations (Fig. 2). Richness was higher in the hard palate microbiota (*P* < 0.05) compared to all other location metagroups except the nostrils; conversely, richness was lowest in the lung, followed by the tonsils (*P* < 0.05) (Additional file 6). The hard palate also had the greatest diversity (Shannon diversity index, *P* < 0.05), while the oral location metagroups (excluding the tonsils) in general had higher diversity compared to the other location metagroups (Additional file 7). Diversity in the nostrils and nasopharynx did not significantly differ from the tonsils or lung (*P* ≥ 0.05).

**Fig. 3:**
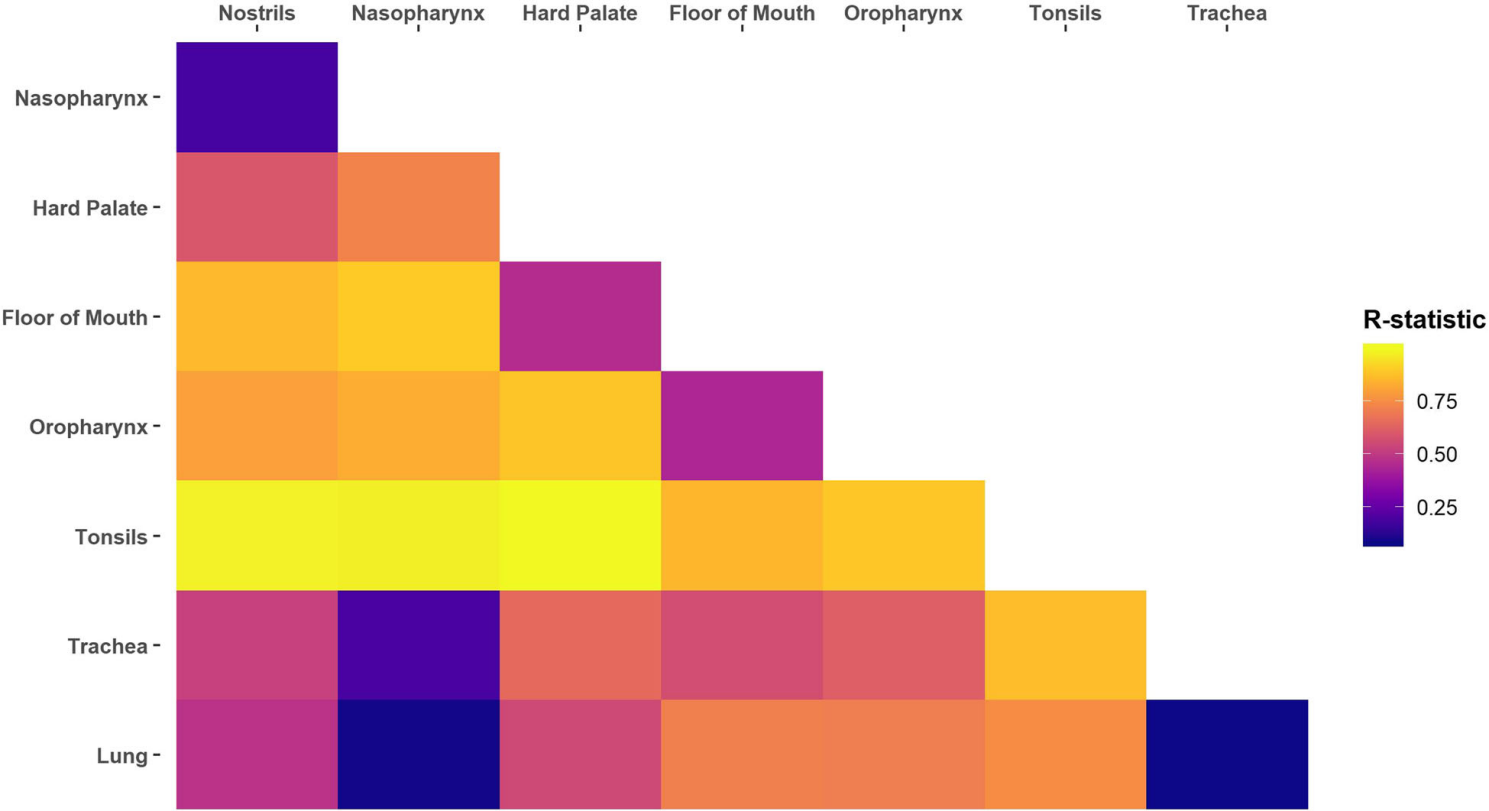
Analysis of similarities results for pairwise comparisons of different upper and lower respiratory sampling location metagroups for 15 healthy beef steer calves

Community composition was dissimilar for the analysis of similarities (ANOSIM) between all location metagroups (*P* < 0.05) except for between the trachea and lung (*P* = 0.137) (Additional file 8). Interestingly, after correcting for multiple comparisons, the nasopharynx and lung were not dissimilar (*P* = 0.082). In support of this observation, the lung microbiota was more similar to the nasopharynx (R-statistic = 0.091) than it was to the oropharynx (R-statistic = 0.709) or any other URT location metagroup (Fig. 3).

**Fig. 4:**
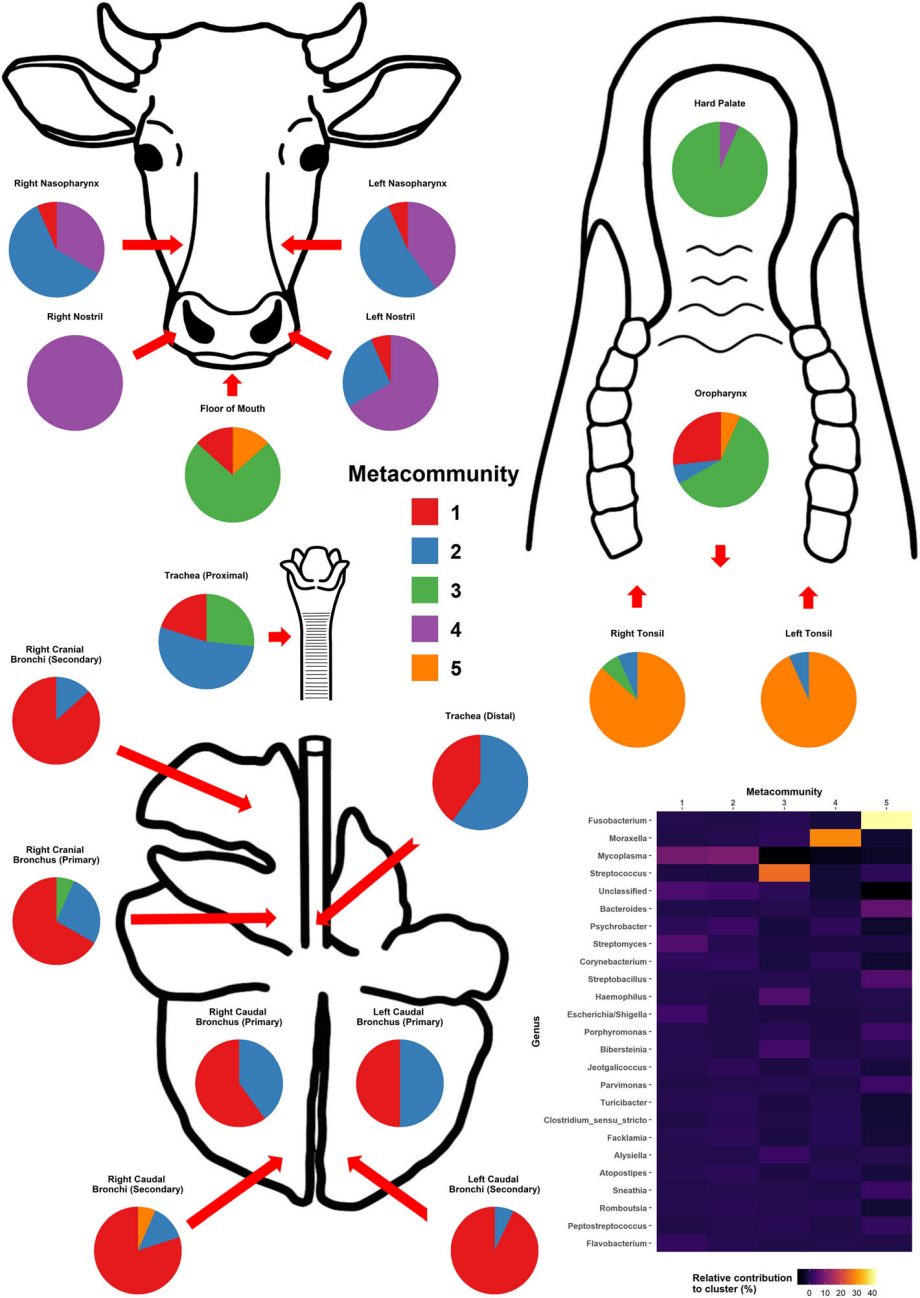
Distribution by respiratory sampling location of five distinct Dirichlet components (metacommunities) clustered at the genus level for 15 healthy beef steer calves and the relative contributions of different genera to each metacommunity

Five distinct metacommunities were identified after clustering at the genus level (Additional file 9). Metacommunity 1, which was dominated by *Mycoplasma* (8.58% relative contribution) was found primarily in the lung (specifically in the secondary bronchi), though it was also found in the nostrils, nasopharynx, oropharynx, floor of the mouth, and trachea (Fig. 4). Metacommunity 2, which was also dominated by *Mycoplasma* (9.83% relative contribution), was primarily found in both the lung and nasopharynx sampling locations. Separate clustering at the SV level showed that the metacommunity shared by the lung and nasopharynx was dominated by *M. dispar* (Additional files 10 and 11). Comparatively, metacommunity 3, which was dominated by *Streptococcus* (25.61% relative contribution), was found primarily in the oropharynx, hard palate, and floor of the mouth, while metacommunity 4, which was dominated by *Moraxella* (28.87% relative contribution), was found primarily in the nostrils. Metacommunity 5, which was dominated by *Fusobacterium* (41.28% relative contribution), was found primarily in the tonsils.

## Discussion

This study was the first to describe and compare the bacterial microbiota of different niches present along the entire respiratory tract of healthy cattle and determined which URT microbiota is most similar to the composition of the lung microbiota. We showed that community composition and diversity differed among different niche microbiota. These differences were driven by a variety of taxa, notably *Mycoplasma*, *Moraxella*, *Streptococcus*, and *Fusobacterium*. We also showed that the lung microbiota was more compositionally similar to the nasopharynx than any other URT microbiota, including the nostrils, oropharynx, and tonsils. This finding indicates that the nasopharynx is likely the primary source of bacteria for the lung in healthy cattle.

The characterization of distinct niche microbiota throughout the bovine respiratory tract is a novel finding. Compositional differences between URT and lower respiratory tract (LRT) microbiotas have been previously observed in cattle [13–15]. However, these past studies have been limited in scope, comparing only trans-tracheal aspirations or bronchoalveolar lavages to the nasopharynx. The findings of the current study corroborate what has been seen in humans and other ruminants. Previous research has shown humans to have marked dissimilarity between the nasal, oral, and lung microbiotas [7, 16, 17]. As well, the microbiota of the oral cavity in lambs has been reported to be distinct from the lung [18].

The presence of distinct niche microbiota across the respiratory tract was expected as there are known physiological and biochemical differences among the many different respiratory locations. Spatial heterogeneity in pH, CO_2_ levels, temperature, epithelial cell types, mucosae thickness, and immune cells have been found throughout the respiratory tract, with human studies even showing heterogeneity in different regions of the lungs [17, 19–22]. The URT is also under constant external pressure from the surrounding environment, which can significantly impact community composition [22].

Despite the characterization of numerous distinct microbiotas throughout the respiratory tract, noticeable compositional overlap was still observed between various niches. Interestingly, though there was significant variation in bacterial composition between sampling locations, there was limited variation across lung sites or between anatomically similar sampling locations (i.e., left and right nostrils, left and right nasopharynx, left and right tonsils). Research in humans has shown that there is little spatial variation across different lung sites within healthy individuals [17]. Yet, in sheep it was found that, depending on the individual animal, there may or may not be significant variation between lung sites [21].

The lack of bacterial variation that we observed within the lung may be partially explained by the adapted island model of lung microbiota biogeography [19]. In this model, the bacterial composition of the lung is determined more by the constant flow of transient bacteria than the replication of resident bacteria [17, 23]. Foreign bacteria are constantly migrating into the respiratory tract through a combination of inhalation, aerosolized saliva microaspiration, and dispersion along mucosal surfaces [19, 24–26]. At the same time, these bacteria are also being constantly cleared from the respiratory tract by forced exhalation (coughing/sneezing) and host respiratory defenses (i.e., mucociliary clearance, antimicrobial peptides, immunoglobulin A, and immune defense cells) [26, 27]. This model concept supports our finding that compositional overlap between URT and LRT microbiotas occurred, and that the URT microbiotas were a source of bacteria residing in the lungs of healthy cattle. It should be noted that this model may not apply to sick animals, as it has been shown that the progression of lung diseases (such as cystic fibrosis) can impair bacterial clearance, leading to increased colonization and proliferation of bacteria in the lung [28, 29]. Regardless, understanding which URT microbiotas contribute the most to the healthy lung is a key component in understanding the complexities of the respiratory system in cattle.

Contrary to what has been seen in humans, the oral microbiotas (specifically the oropharynx) have less compositional overlap with the lung than the nasopharynx. It has been proposed that, in healthy adult humans, aerosolized saliva containing bacteria are aspirated into the lungs during sleep as a result of the throat muscles relaxing [7, 23]. While the oropharynx has been suggested as the primary source of bacteria to the lung in healthy adult humans, it has also been suggested that the oropharynx and nasopharynx both contribute to the lung microbiota in healthy neonates [30]. This process may not be the same in cattle though, as ruminants have notable differences in respiratory anatomy and physiology compared to humans. A study performed in lambs compared the oropharyngeal and ruminal microbiotas with the lung and found that they were significantly dissimilar [18]. These differences might be explained by the horizontal disposition of the lung or by evolutionary anatomical barriers to microaspiration of ruminal fluid into the lung [18]. It is also worth noting that the bovine trachea is longer relative to body size compared to other animals, which may also add to the differences observed between human and ruminant respiratory microbial compositions [27].

The findings of this study have significant implications for the field of respiratory biology in cattle. Notably, the nasopharynx appears to be the most important location that should be targeted when doing respiratory microbiota research. As well, these results suggest that the nasopharynx may be the optimal microbiota to target for novel therapeutics aimed at promoting respiratory health.

It is possible that the oral microbiotas in cattle act as secondary bacterial reservoirs for the lung. The oral microbiotas were primarily dominated by the genus *Streptococcus* in the current study, corroborating what has been previously seen [31, 32]. Interestingly, we were able to identify a metacommunity dominated by *Streptococcus* in the oral microbiotas as well as the lung, though we were unable to identify most of the bacteria in this genus at the species level. Greater clarity at the species level could be valuable, as overlap between the ruminal and oral microbiotas has been previously observed [33]. The process of rumination, whereby cattle regurgitate feed to be masticated a second time before swallowing the feed again, is integral to the digestion in cattle [33]. *Streptococcus bovis*, a known rumen inhabitant that plays an increased digestive role in cattle that are being transitioned to high starch diets, has the potential to gain entry to the oral cavity via rumination [33–35]. In the current study, cattle were in the process of transitioning to a high starch diet (i.e., corn-based) at the time of sampling, providing a potential explanation for the observed abundance of *Streptococcus* in the oral microbiotas. Whether the *Streptococcus* observed in the oral and lung microbiotas in this study were *S. bovis* or a different *Streptococcal* species is unclear, and further research is needed to understand the relationships between the ruminal, oral, and lung microbiotas.

The palatine tonsils may also act as a secondary reservoir of bacteria for the lung microbiotas. We observed that the tonsils were dominated by the bacterium *F. necrophorum* in the current study. This finding is notable, as *F. necrophorum* is a normal inhabitant of the rumen and also the primary causative pathogen for liver abscesses in cattle [36]. We were able to identify a metacommunity dominated by *F. necrophorum* in both the tonsillar microbiotas and the lung. This again suggests not only a possible link between these microbiotas but also between the rumen and the lung microbiotas. Additionally, we found at least one of *H. somni*, *P. multocida*, or *Mycoplasma bovis* in the tonsils of each calf, with multiple calves harboring more than one of these pathogens. In contrast, *M. haemolytica* was only found in the tonsils of one calf. Previous research has shown that tonsils inoculated with *M. haemolytica* can serve as a reservoir for the bacterium [8, 37–39]. During periods of stress or respiratory viral infection *M. haemolytica* can be shed from the tonsils [37–39]. However, in healthy calves, it has been shown that *M. haemolytica* shed from the tonsils into the nasal mucous are rapidly cleared from the nasal passages [8, 38, 39]. As we only studied healthy animals, we cannot rule out the tonsillar microbiota as a potential source of bacterial pathogens for the lung.

The high abundance of *Moraxella* in the nasal passageways observed in the current study agreed with findings from previous research. *Moraxella* is often found to be one of the most abundant genera in the URT of cattle [14, 40–42]. The role of this genus in BP has been brought into question before, with one previous study finding an association between *Moraxella* and the development of pneumonia and/or otitis in the early life of dairy calves [43]. It is also known that *M. bovoculi* and *Moraxella bovis* are opportunistic pathogens, commonly accepted as being the primary etiological agents of infectious bovine keratoconjunctivitis (IBK) [44, 45]. In contrast, another study found that *M. bovoculi* was one of the top bacterial species driving differences in community composition between healthy cattle and those that had developed BP, with a higher abundance of the bacterium found in the nasopharynx of healthy calves [40]. Thus, the strain of *Moraxella* may determine pathogenicity and subsequent roles, if any, in BP. Indeed, it has been shown that *M. bovoculi* isolated from the eyes of cattle that had developed IBK were significantly different at a genomic level from nasopharyngeal isolates found in healthy cattle [46]. That we found a high abundance of *Moraxella*, more specifically *M. bovoculi*, in both the nostrils and nasopharynx of healthy cattle might suggest that certain strains of these bacteria may be part of the normal, healthy nasal microbiota. Future research using whole-genome sequencing techniques could provide greater clarity at lower levels of taxonomic identification on what role *Moraxella* may play in cattle respiratory health.

*Mycoplasma*, specifically *M. dispar*, was frequently identified in both the lung and nasopharynx, with the highest relative abundances observed in the lung. This finding echoes a number of other studies that have reported *Mycoplasma* as one of the most commonly identified genera in the nasopharynx and lung [13, 14, 40–42, 47]. It is not clear what role *M. dispar* plays in respiratory health. This bacterium has been previously isolated from the lungs of both healthy and pneumonic cattle [48–50]. Interestingly, a study by Timsit et al., 2018 identified a distinct metacommunity, which was characterized by an over-representation of *M. dispar* (and other commensal bacteria such as *Lactococcus lactis* and *Lactobacillus casei*) in the lungs of healthy feedlot cattle. While *M. dispar* has been shown to have a number of virulence factors for bovine epithelial cells and can have immunosuppressive effects, it is associated with only milder respiratory infections and is likely not a causative agent for BP [48, 50, 51]. In general, *Mycoplasma* has a high affinity for binding to respiratory epithelial cells via adhesin proteins [52, 53]. That *M. dispar* elicits a milder cellular response might explain why we found this bacterium in such high abundance in the lungs of healthy calves compared to more pathogenic *Mycoplasma*. A previous study that compared two genetically similar *Mycoplasma* species, *Mycoplasma hyopneumoniae* (a causative agent of porcine enzootic pneumonia) and *Mycoplasma flocculare* (a regular commensal bacterium in the respiratory tract of swine), found that differences in orthologous surface proteins were associated with distinct immunological responses, potentially affecting bacterial survivability [54]. Whether *M. dispar* confers some form of protective effect to the host (such as inhibiting *Mycoplasma bovis* colonization in healthy cattle by competing for adhesion sites) or is simply a common respiratory commensal bacterium remains to be determined. It would be valuable to understand when and how the nasopharynx and lungs of healthy calves are colonized by *M. dispar*, as previous studies have reported increased abundance/isolation of the bacterium over the first half-year of life and notably after weaning [49, 55].

There were several strengths to this study. The potential for cross contamination between the upper and lower respiratory sampling locations was limited by using two endoscopes and custom protected specimen brushes (PSBs) with a cellulose plug and triple-sheathed catheters. The sample collection, DNA extraction, and DNA amplification/sequencing techniques employed were reliable and did not introduce significant contamination, as supported by the negative and positive control samples. Strong health status criteria were used to select calves for study enrollment, including a clinical examination by a trained veterinarian, measuring rectal temperature, performing a lung ultrasonography and thoracic auscultation, and determining serum haptoglobin concentration. As well, DADA2 was used to infer exact SVs with resolution at the single nucleotide level, allowing for a more in-depth and accurate analysis of the different niche microbiota in comparison to the standard method of binning sequences into operational taxonomic units (OTUs) by 97% similarity. However, there were also limitations to the study. Only healthy calves were enrolled, for whom pathogen abundance and presence were expectedly low; thus, we could not comment on sampling location similarities or differences as they relate to BP. We also did not test for specific BP pathogens (i.e., through culturing or pulsotyping), so we cannot be certain that other niche microbiota (such as the tonsils) do not contribute bacterial pathogens to the lung. Only newly arrived (less than a year old) beef feedlot steers from one feedlot were studied, preventing us from commenting on community similarities or differences in cattle of different ages or from different geographic locations. While we were able to identify SVs at the species level, many could not be classified; accordingly, all species-level results should be interpreted with caution. Finally, we only looked at calves that had never received an antimicrobial at any point during their life, limiting our understanding of how antimicrobials may affect different respiratory microbiota.

## Conclusions

The nasopharyngeal bacterial microbiota is most similar to the lung bacterial microbiota in healthy cattle and therefore may serve as the primary source of bacteria to the lung. This finding indicates that the nasopharynx is likely the most important location that should be targeted when doing bovine respiratory microbiota research.

## Methods

### Study animals

Candidate animals for this study were recently weaned, crossbred beef-breed feedlot steer calves that were raised without the use of antimicrobials since birth (i.e., “natural” program). Calves arrived at the feedlot directly from calf-ranches in January 2019. A total of 18 steers were enrolled to the study over three days in February 2019. As no prior data were available on the relative contributions of the nasopharyngeal and oropharyngeal microbiotas as source communities to the lung microbiota in cattle, it was not possible to calculate an a priori sample size. The sample size was therefore based on availability of cattle at the feedlot and costs, with a minimum of 15 cattle.

On arrival at the feedlot, steers were processed according to standard feedlot protocols; they received a topical avermectin (Bimectin™, Bimeda-MTC Animal Health Inc., Cambridge, ON, Canada), a clostridial vaccine (Ultrabac® 7/Somubac®, Zoetis Canada Inc., Kirkland, QC, Canada), and a multivalent modified live viral vaccine with a *M. haemolytica* toxoid (Pyramid® FP 5 + Presponse® SQ, Boehringer Ingelheim (Canada) Ltd., Burlington, ON, Canada).

Subsequent animal husbandry followed standard feedlot protocol, as previously described [40]. After arrival processing, study steers were commingled with other steers and housed in large, outdoor dirt-floor pens with capacities between ~ 250 and 300 cattle/pen. Twice daily steers were fed a corn-based diet formulated to meet or exceed nutrient requirements. This diet did not contain any in-feed antimicrobials. Feed bunks were visually inspected and evaluated every day prior to feeding, and feed deliveries were adjusted accordingly to ensure that steers had access to sufficient feed to allow for *ad libitum* consumption.

### Study design

Apparently healthy steers were conveniently selected by feedlot staff and presented to the feedlot hospital facility on the day of study enrollment for clinical evaluation. Once it was determined that there had been no prior treatment for clinical BP or other diseases during the feeding period, an experienced study veterinarian (ET) examined each steer for inclusion to the study. This included a visual assessment of the steer for clinical signs associated with BP, specifically depression, cough, nasal discharge, and ocular discharge. A complete thoracic auscultation was also performed to detect abnormal lung sounds, as described [56]. Furthermore, a thoracic ultrasonography of the cranio-ventral portion of both sides of the thorax was performed to detect lung consolidation (> 1 cm deep) or pleural effusion, as described [57]. Rectal temperature was also measured. Finally, whole blood was collected using plain tubes (BD Vacutainer® Rapid Serum Tube, BD Canada, Mississauga, ON, Canada) for haptoglobin analysis, as described [58].

Steers that did not exhibit any visual signs associated with BP and that had normal lung sounds, no lung consolidation (> 1 cm deep) or pleural effusion detected at thoracic ultrasonography, and a rectal temperature < 40 °C were sampled as described below. Only samples coming from steers that had a serum haptoglobin concentration ≤ 0.25 g/L were further analyzed [58].

### Sampling procedures and sample processing

A total of 17 respiratory tract locations were sampled for each steer. This included the left and right nostrils, left and right nasopharynx, hard palate, floor of the mouth (area under the tongue), oropharynx (soft palate), left and right palatine tonsils, trachea (proximal and distal), left caudal bronchus (primary), left caudal bronchi (secondary), right caudal bronchus (primary), right caudal bronchi (secondary), right cranial bronchus (primary), and right cranial bronchi (secondary). The proximal trachea sample was collected from the tracheal mucosae immediately distal to the larynx. The distal trachea sample was collected from the tracheal mucosae immediately proximal to the carina.

The first locations sampled from each steer were the nostrils followed by the nasopharynx. Briefly, a paper towel was used to thoroughly wipe out both nostrils from each steer in order to remove potential debris. A short, flocked nylon fiber tip swab (9 cm long; BD ESwab™ Collection Kit Regular Flocked Swab, BD Canada) was inserted into the left nostril and vigorously moved back and forth against the mucosal surface. The swab was then removed from the steer’s nostril and inserted into a transport tube containing liquid Amies transport media (all swab samples were stored in the same type of transport tube). This process was repeated for the right nostril. Next, a long, guarded swab with a rayon tip (27 cm long; MW 124, Medical Wire & Equipment, Corsham, United Kingdom) was inserted into the left nostril, down into the nasopharynx, as described [40]. The nasopharynx was sampled by extending the swab beyond the guard and vigorously moving it back and forth against the mucosal surface. After retracting the swab behind the guard, the entire swab was removed from the steer’s nasal passageway. The swab was then extended beyond the guard and the tip inserted into a transport tube, where it was removed from the rest of the swab using scissors. This process was repeated for the right nasopharynx.

The next locations sampled were the hard palate and floor of the mouth using short, flocked nylon fiber tip swabs. Briefly, the steer’s mouth was held open and its tongue held to the side by hand. A short, nylon fiber tip swab was moved back and forth against the hard palate. The swab was then removed from the steer’s mouth and inserted into a transport tube. This process was repeated for the floor of the mouth, with the swab moved back and forth against the mucosal surface under the tongue.

Next, to collect samples from the tonsils and oropharynx, a long, hollow metal tube was inserted into the mouth of the steer. A 105 cm long video endoscope was passed through the tube along with a double-guarded swab with a polystyrene cotton tip (84 cm; J0273, Jorgensen Laboratories, Inc., Loveland, CO, USA), with the swab retracted behind the guards. After the left tonsil was located using the camera in the endoscope, the swab was extended beyond the guards directly into the tonsil, where it was moved back and forth against the tonsillar lymphatic tissue (Additional file 12). After retracting the swab behind the guard, the entire swab was removed from the steer’s oral passageway. The swab was then extended beyond the guard and the tip inserted into a transport tube, where it was removed from the rest of the swab using scissors. This process was repeated for the right tonsil. The oropharynx was then sampled using a similar method, with the swab being moved vigorously back and forth against the mucosal surface of the soft palate.

Using a 140 cm long video endoscope, the trachea and primary bronchi of the lung were sampled next. The video endoscope was inserted into the left nostril and down through the nasal passageway to just beyond the larynx. A double-guarded PSB (custom-made, gas sterilized; brush diameter 3.0 mm, length 200 cm [00109, ConMed Canada, Mississauga, ON, Canada] protected by an additional outer Teflon sheath with a cellulose plug at the distal end [custom-made by Mila International Inc., Florence, KY, USA]) was passed through the channel of the video endoscope until it protruded into the trachea. The cellulose plug was pushed out of the catheter using the specimen brush (Additional file 13), which was extended into the respiratory tract and moved back and forth against the mucosal surface of the proximal trachea (Additional file 14). After retracting the brush behind the protective catheter, the entire apparatus was removed from the endoscope. The endoscope was then pushed deeper into the respiratory tract, with the same process repeated for the right cranial bronchus, distal trachea, left caudal bronchus, and right caudal bronchus, in that order. After collection, each PSB was extended beyond the protective catheter and the tip inserted into a transport tube containing liquid Amies transport media, where it was removed from the rest of the apparatus using wire cutters.

Without removing the video endoscope used for the PSB samples, a triple-sheathed catheter was passed through the endoscope (EMAC800, Mila International Inc.) and down into the primary left caudal bronchus. Using a sterile 30-mL syringe, ~ 10 mL of sterile saline was pushed into the secondary bronchi of the right caudal lobe. The saline was recovered by aspirating the syringe repeatedly until ~ 2 mL of fluid was obtained. This process was repeated for the left caudal and then right cranial secondary bronchi. Saline was transferred via needle to individual sterile 4 mL plain tubes (BD vacutainer rapid serum tubes).

At the time of study sample collection, negative control samples (n = 13) were collected for the different swabs and brushes outside the animals. As well, negative control samples (n = 6) were collected from five different study animals to assess the risk for potential contamination when passing the endoscope down through the nasal passageway into the lower respiratory tract (one animal was sampled twice due to possible contamination of the first sample collected; the contaminated sample was removed from the study after sequencing data were processed and the sample was assessed). These samples were collected using a PSB by extending the brush into the distal trachea without touching the brush to any surface and retracting the brush behind the protective catheter.

All samples were immediately stored in a polystyrene cooler on ice packs and transported within 6 h to the University of Calgary, Calgary, AB. After arrival at the university, swabs and brushes were removed from their transport tubes and placed into individual 1 mL aliquots of 20% glycerol/80% brain heart infusion (BHI) broth in microcentrifuge tubes. The transport tubes were centrifuged at 2000×*g* for 5 min. The supernatant was removed, and the pellets were resuspended in 0.2 mL of 20% glycerol/80% BHI broth and added to the tube with their respective swab/brush. The saline wash samples were also centrifuged at 2000×*g* for 5 min. The supernatant was removed, the pellets were resuspended in 1.2 mL aliquots of 20% glycerol/80% BHI broth, and the resuspended pellets were placed in individual microcentrifuge tubes. Each microcentrifuge tube was vortexed for 30 s. Samples were then frozen at −80 °C until laboratory diagnostic work was performed.

### DNA extraction

A commercially available extraction kit (DNeasy® Blood & Tissue Kit, QIAGEN Inc., Mississauga, ON, Canada) was used to extract total DNA from all samples, as previously described [40]. Briefly, the swabs and brushes were removed from each sample tube and placed in individual sterile tubes on ice. The sample tubes containing glycerol/BHI broth, including those from which the swabs/brushes were removed and those containing a resuspended pellet from the saline wash samples, were centrifuged at 5000×*g* for 5 min. Supernatant was pulled out of each sample tube and discarded. The swabs/brushes were then returned to their original respective tubes. The swabs/brushes and pellets were then suspended in 180 μl of an enzymatic lysis buffer containing lysozyme (100 mg ml^−1^) and mutanolysin (25,000 U ml^−1^). Each sample mixture was vortexed at 300 rpm and incubated at 37 °C for 1 h. Next, an ethanol-free lysis buffer (200 μl) and proteinase K (25 μl) were combined with each mixture. Each sample mixture was individually vortexed, and then incubated for 30 min at 56 °C. Approximately 300 mg of sterile 0.1 mm zircon/silica beads were poured into each sample mixture and beaten using a TissueLyser LT (QIAGEN Inc.) for 5 min at 30 Hz. Each mixture was then centrifuged at 13,000×*g* for 5 min and the resulting supernatant was transferred to new individual sterile microcentrifuge tubes. The supernatant was combined with ethanol (200 μl) and each tube was vortexed. The DNeasy Blood & Tissue Kit protocol, in accordance with the instructions provided by the manufacturers, was used from this point forward to finish the extraction process. DNA extractions performed within the same day used the same reagents, and all reagents came from one kit. A negative control sample was included for each day of extractions, which involved the DNA extraction steps outlined above, minus the presence of sample material (n = 11 negative controls). Positive control samples were included to assess both the DNA extraction process and subsequent targeted amplicon sequencing (n = 2 positive controls). Each positive control consisted of a bacterial mock community comprised of a 10-strain mix of whole-cell material (ATCC® MSA-2003™, Manassas, VA, USA).

### Amplification and sequencing

Targeted amplicon sequencing (16S rRNA gene) for all DNA samples was performed at Génome Québec, located in Montréal, QC, Canada, as previously described [40]. The V3-V4 hypervariable regions of the 16S rRNA gene were amplified using primers 341F (ACACTGACGACATGGTTCTACA) and 805R (TACGGTAGCAGAGACTTGGTCT). Each primer was modified to include adapters designed to bind DNA to a flow cell for sequencing, as well as index barcodes to allow for library multiplexing. DNA was amplified using 25 μL reaction mixtures that contained each primer at a concentration of 0.6 μM, deoxynucleoside triphosphate at a concentration of 0.2 mM, dimethyl sulfoxide at a concentration of 5%, TAQ 5 U-μl polymerase at a concentration of 0.02 U/μL, 2.5 μL of 10X polymerase chain reaction (PCR) buffer with 18 mM of MgCl_2_, 19.35 μL of distilled water, and 1 μL of DNA. Amplification via PCR entailed an initial denaturation step at 94 °C for 2 min. Initial denaturation was followed by 33 cycles of 94 °C for 30 s, 60 °C for 30 s, and 72 °C for 30 s, finishing with an extension step at 72 °C for 7 min. Verification of DNA barcoding and amplification were performed separately on 2% agarose gels. A Quant-iT™ PicoGreen® dsDNA Assay Kit (Thermo Fisher Scientific, Waltham, MA, USA) was used to quantify total DNA amplified.

DNA libraries were set up by pooling 25 ng of individual samples together. All libraries were cleaned with sparQ PureMag Beads (Quantabio, Beverly, MA, USA). A Quant-iT™ PicoGreen® dsDNA Assay Kit (Life Technologies, Burlington, ON, Canada) was used to quantify each amplicon. A universal KAPA Library Quantification Kit for Illumina® Platforms with Revised Primers and Kapa SYBR® Fast (Kapa Biosystems, Wilmington, MA, USA) was used to quantify the libraries. Average fragment size was established using a LabChip GX instrument (PerkinElmer, Waltham, MA, USA). In order to ameliorate unbalanced base composition, 10% of the PhiX control library was added to the amplicon pool (final loading concentration of 10 pM) prior to DNA sequencing. A MiSeq Reagent Kit v3 (600 cycles) (Illumina, Inc., San Diego, CA, USA) was used according to the instructions provided by the manufacturer to perform 16S rRNA gene amplicon sequencing. Additionally, LNA™ modified custom primers (Exiqon, Copenhagen, Kingdom of Denmark) were included in the amplicon sequencing process (Primer read 1 – ACACTGACGACATGGTTCTACA; primer read 2 – TACGGTAGCAGAGACTTGGTCT; Primer index read – AGACCAAGTCTCTGCTACCGTA). Following sequencing, Génome Québec demultiplexed the libraries and removed all adapters and index barcodes from the sequence data.

### Sequence processing

Sequencing data were processed as previously described [40] using cutadapt v2.3 [59] and DADA2 v1.10 [60] as implemented in R v3.5.1 [61]. Forward and reverse 5’ 16S primers, as well as low-quality ends, were trimmed from the raw sequencing data using cutadapt in paired-end mode with a maximum allowed error rate of 0.1 and a quality cutoff of 20. Reverse compliment primers were not trimmed as the targeted read length was 2 × 300 base pairs; as the approximate length of the V3-V4 regions is 460 base pairs, reverse compliments of the forward and reverse 5’ primers were never sequenced and therefore were not present in the data. Sequencing data quality was then assessed using FastQC v0.11.8 [62]. Individual sample quality reports were compiled into one comprehensive report using MultiQC v1.7 [63]. Once the quality of the data was deemed acceptable, DADA2 was utilized to filter and trim reads, infer exact SVs, and assign taxonomy to SVs. Default parameters were used for all DADA2 functions unless expressly mentioned. Reads were filtered using a maximum expected error of one. A parametric error model was then estimated through a form of unsupervised machine-learning. This estimation was performed using 100 million sequences each for the forward and reverse reads separately. Sequencing reads were then dereplicated. Exact amplicon SVs were inferred for each sample using the DADA2 sample inference algorithm and the estimated error model. Samples were pooled together for sample inference to increase sensitivity to SVs present at extremely low frequencies. Full, denoised sequences were obtained by merging the inferred forward and reverse reads. An SV table, which is functionally similar to an operational taxonomic unit table, was assembled from the denoised sequences. Chimeric sequences were then removed from the table. A taxonomy table was assembled by assigning taxonomy to each SV in the SV table using the RDP [64] taxonomic database for DADA2 [65]. All species-level assignment was accomplished using the DADA2::addSpecies function, with exact matching used to assign species when possible.

### Statistical analyses

Downstream analyses were performed in R using multiple functions from phyloseq v1.26.1 [66], ggpubr v0.2.4 [67], RVAideMemoire v0.9-74 [68], vegan v2.5-6 [69], and DirichletMultinomial v1.28.20 [70]. An object was constructed from the SV and taxonomy tables in R using phyloseq for subsequent analysis. A prevalence filter was applied to the phyloseq object such that only SVs present in ≥ 1% of the samples remained. Mean relative abundance and beta-diversity measures were calculated using prevalence filtered data; alpha-diversity measures were calculated using unfiltered data.

To facilitate downstream analyses, samples were classified into location metagroups based on preliminary analyses and anatomical/functional similarity among sampling locations. These metagroups included the nostrils (left and right nostrils), nasopharynx (left and right nasopharynges), hard palate, floor of the mouth, oropharynx, tonsils (left and right tonsils), trachea (proximal trachea), and lung (everything from the distal trachea down into the lung). All preliminary analyses incorporated pairwise comparisons of the different sampling locations and included comparisons of select alpha-diversity metrics, a permutational multivariate analysis of variance (PERMANOVA), and an ANOSIM, as detailed below. Preliminary results are included as supplementary files (Additional files 15 and 16).

Species richness (Chao1 index) and diversity (Shannon diversity index) were calculated for all samples as implemented in phyloseq. Pairwise comparisons of alpha-diversity measures were made using Wilcoxon rank-sum tests to compare alpha-diversity group means between sampling locations and location metagroups as implemented in ggpubr. Adjustments for multiple comparisons were made using the Holm method.

A pairwise ANOSIM using a Bray-Curtis dissimilarity index as implemented in the vegan package was used to evaluate compositional similarities between different sampling locations and location metagroups. An individual ANOSIM was performed for each pairwise sampling location comparison and all *p* values were adjusted for multiple comparisons using the Benjamini & Hochberg method as implemented in vegan. A similar procedure was used to compare location metagroups, with all *p* values adjusted for multiple comparisons using the Holm method as implemented in vegan.

A pairwise PERMANOVA using a Bray-Curtis dissimilarity index as implemented in the RVAideMemoire package was used to evaluate compositional differences between different sampling locations. The RVAideMemoire package corrected for multiple comparisons using the Benjamini & Hochberg method. To visualize these compositional differences, data were ordinated using non-metric multidimensional scaling (NMDS) and a Bray-Curtis dissimilarity index as implemented in phyloseq.

Finally, samples were clustered using Dirichlet multinomial mixtures [71] as implemented in the DirichletMultinomial package, as previously described [13]. Using a Laplace approximation, the number of Dirichlet components (i.e., metacommunities) that fit the data best was determined. Contributions of different taxa to each metacommunity were determined by comparing the best fit model to one with a single metacommunity as described [70]. Individual taxa were assigned to the metacommunity where it had the highest contribution. Clustering was performed separately at both the genus and individual SV level.

## Supplementary information

**Additional file 1: Spreadsheet S1.** Raw abundance of sequence variants for negative and positive control samples.

**Additional file 2: Table S1.** Mean relative abundance of bacteria present at ≥1% abundance (phylum, genus, and species level).

**Additional file 3: Fig. S1.** Mean relative abundance of bacteria present at ≥1% abundance (phylum level – all sampling locations).

**Additional file 4: Fig. S2.** Mean relative abundance of bacteria present at ≥1% abundance (genus level – all sampling locations).

**Additional file 5: Fig. S3.** Mean relative abundance of bacteria present at ≥1% abundance (species level – all sampling locations).

**Additional file 6: Spreadsheet S2.** Wilcoxon rank-sum test results comparing species richness (Chao1 index) between different sampling location metagroups.

**Additional file 7: Spreadsheet S3.** Wilcoxon rank-sum test results comparing diversity (Shannon diversity index) between different sampling location metagroups.

**Additional file 8: Spreadsheet S4.** Analysis of similarities results from pairwise comparisons of different sampling location metagroups.

**Additional file 9: Spreadsheet S5.** Relative contributions of different genera to multiple Dirichlet components (metacommunities).

**Additional file 10: Spreadsheet S6.** Relative contributions of different sequence variants to multiple Dirichlet components (metacommunities).

**Additional file 11: Fig. S4.** Ordination of relative abundance data by Dirichlet metacommunity and sampling location using non-metric multidimensional scaling.

**Additional file 12: Video S1.** Palatine tonsil sample collection.

**Additional file 13: Video S2.** Pushing cellulose plug out of protected specimen brush catheter during sampling.

**Additional file 14: Video S3.** Proximal trachea sample collection.

**Additional file 15: Table S2.** Preliminary permutational analysis of variance results for pairwise comparisons of all sampling locations.

**Additional file 16: Spreadsheet S8.** Preliminary analysis of similarities results for pairwise comparisons of all sampling locations.

## Data Availability

All sequencing data were deposited to the National Center for Biotechnology Information Sequence Read Archive under accession number PRJNA596300.

## Abbreviations

BP: Bronchopneumonia
URT: Upper respiratory tract
LRT: Lower respiratory tract
PSB: Protected specimen brush
PCR: Polymerase chain reaction
SV: Sequence variant
PERMANOVA: Permutational analysis of variance
ANOSIM: Analysis of similarities
NMDS: Non-metric multidimensional scaling
