# Supplementary material for: Topography of the respiratory tract bacterial microbiota in cattle
Source: Microbiome. 2020 Jun 10;8:91. doi: 10.1186/s40168-020-00869-y (PMC7288481; doi:10.1186/s40168-020-00869-y)
Supplement: Supplementary file 3 — Additional file 2: Table S1. Mean relative abundance of bacteria present at ≥1% abundance (phylum, genus, and species level). [file 40168_2020_869_MOESM2_ESM.docx]

| **Phylum** | **Relative abundance** | **Genus** | **Relative abundance** | **Species** | **Relative abundance** |
| --- | --- | --- | --- | --- | --- |
| *Proteobacteria* | 27.11% | *Moraxella* | 11.65% | *bovoculi* | 1.49% |
|  |  | *Psychrobacter* | 2.44% | *maritimus/psychrophilus* | 1.15% |
|  |  | *Escherichia/Shigella* | 1.89% | *** | 1.75% |
|  |  | *Bibersteinia* | 1.86% | *-* | - |
|  |  | *Bartonella* | 1.29% | *** | 1.29% |
|  |  | *Haemophilus* | 1.09% | *-* | - |
| *Tenericutes* | 22.38% | *Mycoplasma* | 22.28% | *dispar* | 16.02% |
|  |  |  |  | *bovis* | 1.20% |
| *Firmicutes* | 21.36% | *Streptococcus* | 8.56% | *-* | - |
|  |  | *Clostridium_sensu_stricto* | 1.41% | *celatum/disporicum* | 1.19% |
|  |  | *Romboutsia* | 1.10% | *-* | - |
| *Actinobacteria* | 14.00% | *Streptomyces* | 2.63% | *** | 2.63% |
|  |  | *Corynebacterium* | 1.68% | *-* | - |
| *Fusobacteria* | 9.81% | *Fusobacterium* | 7.48% | *necrophorum* | 7.26% |
|  |  | *Sneathia* | 1.39% | *-* | - |
| *Bacteroidetes* | 5.05% | *Bacteroides* | 1.23% | *-* | - |

* denotes a taxonomic group that could be ambiguously attributed to > 5 different species; species names excluded from table
