# Supplementary figures and images for: Topography of the respiratory tract bacterial microbiota in cattle
Source: Microbiome. 2020 Jun 10;8:91. doi: 10.1186/s40168-020-00869-y (PMC7288481; doi:10.1186/s40168-020-00869-y)

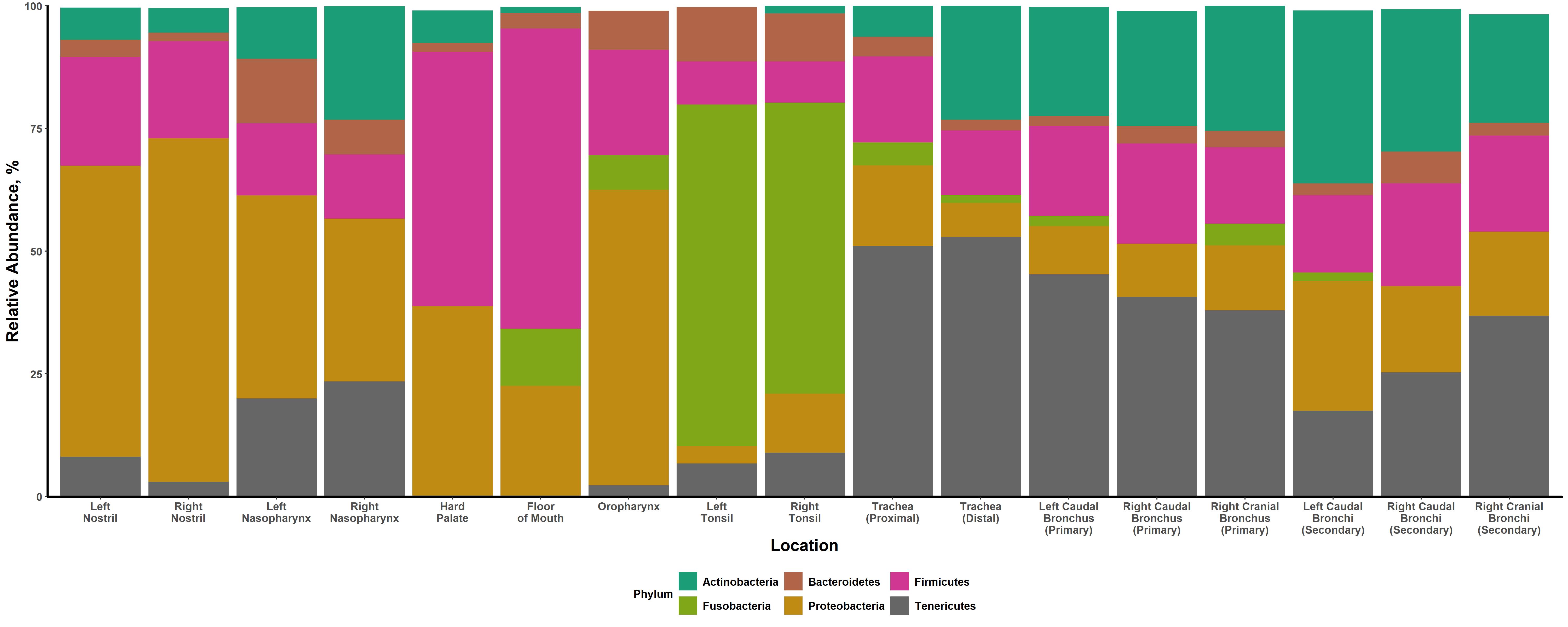

Supplement: Supplementary file 4 — Additional file 3: Fig. S1. Mean relative abundance of bacteria present at ≥1% abundance (phylum level – all sampling locations). [file 40168_2020_869_MOESM3_ESM.jpg]

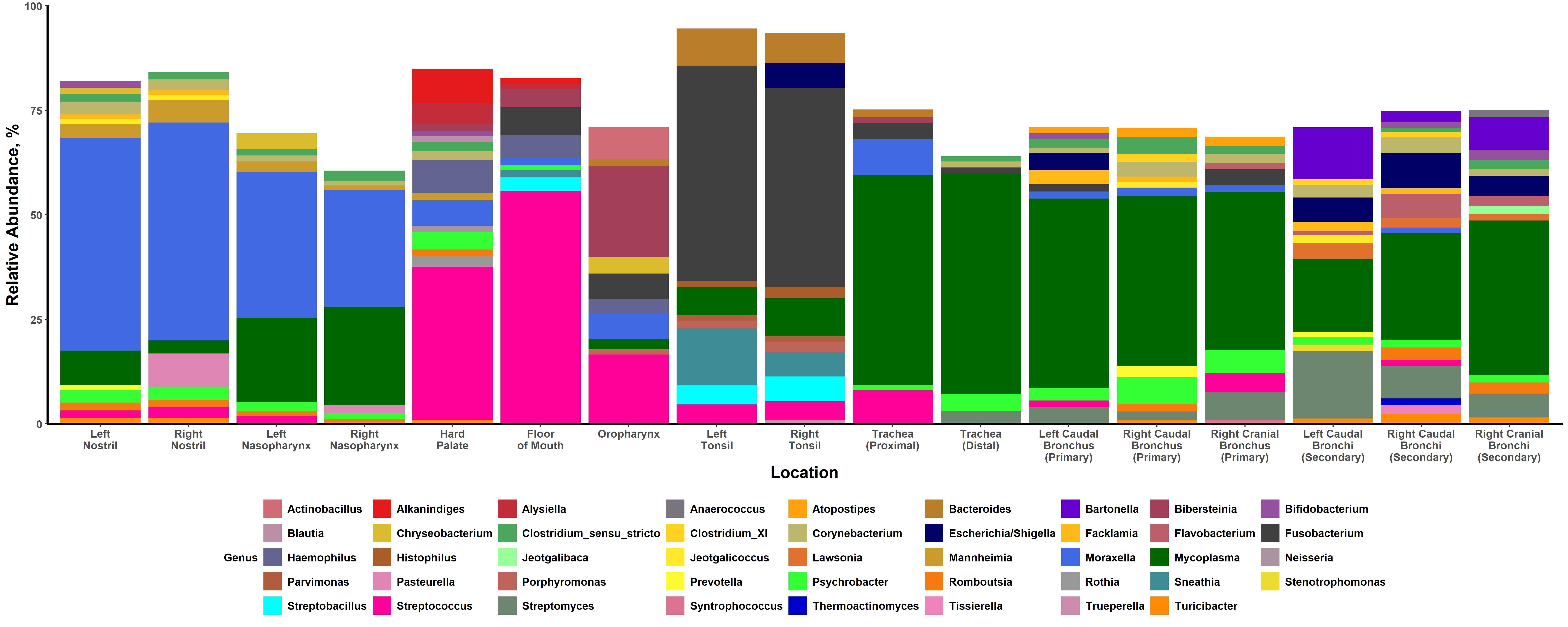

Supplement: Supplementary file 5 — Additional file 4: Fig. S2. Mean relative abundance of bacteria present at ≥1% abundance (genus level – all sampling locations). [file 40168_2020_869_MOESM4_ESM.jpg]

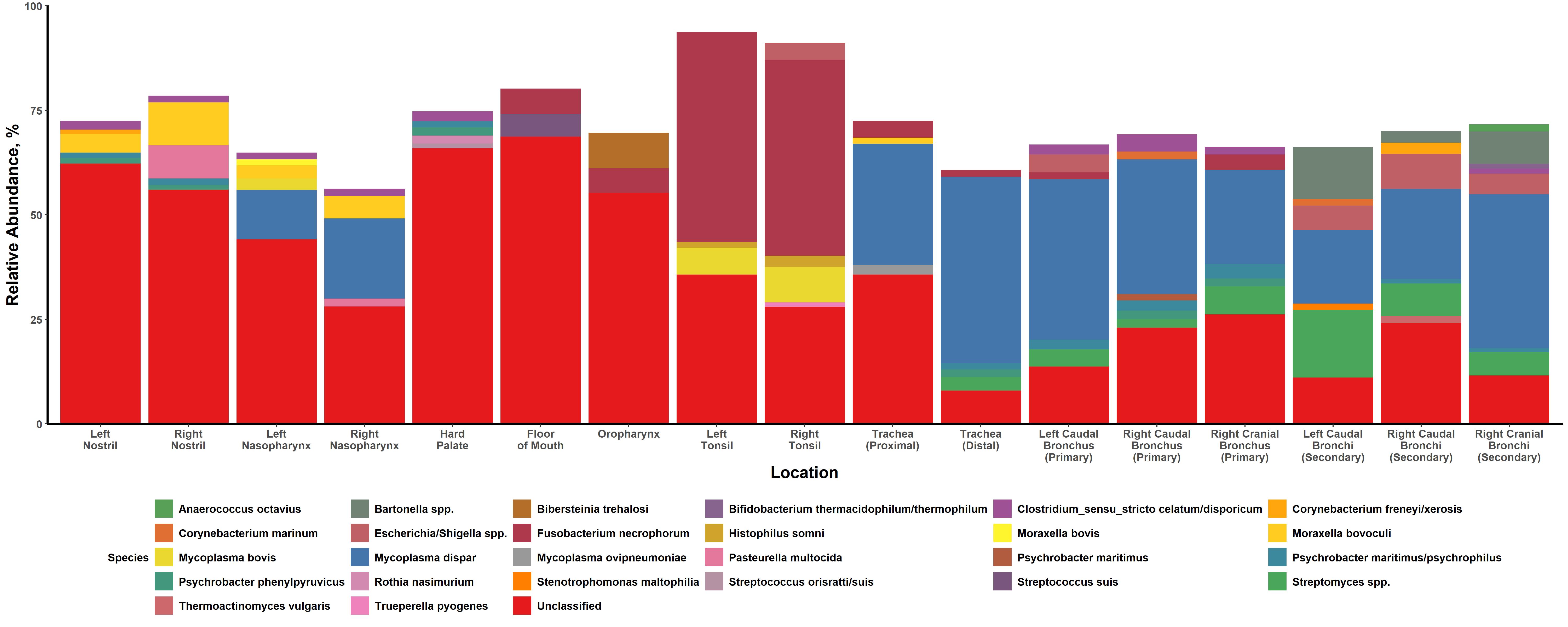

Supplement: Supplementary file 6 — Additional file 5: Fig. S3. Mean relative abundance of bacteria present at ≥1% abundance (species level – all sampling locations). [file 40168_2020_869_MOESM5_ESM.jpg]

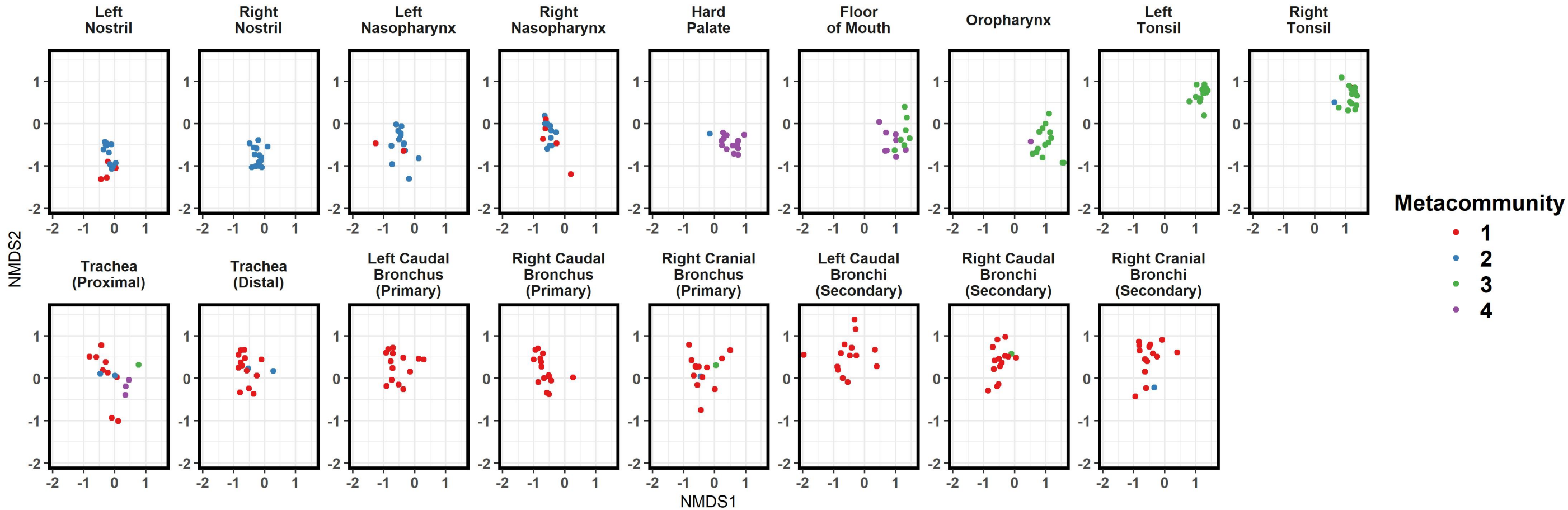

Supplement: Supplementary file 12 — Additional file 11: Fig. S4. Ordination of relative abundance data by Dirichlet metacommunity and sampling location using non-metric multidimensional scaling. [file 40168_2020_869_MOESM11_ESM.pdf]
