# Supplementary material for: Topography of the respiratory tract bacterial microbiota in cattle
Source: Microbiome. 2020 Jun 10;8:91. doi: 10.1186/s40168-020-00869-y (PMC7288481; doi:10.1186/s40168-020-00869-y)
Supplement: Supplementary file 16 — Additional file 15: Table S2. Preliminary permutational analysis of variance results for pairwise comparisons of all sampling locations. [file 40168_2020_869_MOESM15_ESM.docx]

Pairwise comparisons using permutation MANOVAs on a distance matrix

data: distLocation by distLocationdf$Location

999 permutations

BO BT CR LB LCL LN LNP LT OR RB RCB RCL RN RNP RT TO

BT 0.0015 - - - - - - - - - - - - - - -

CR 0.0015 0.1841 - - - - - - - - - - - - - -

LB 0.0015 0.7532 0.6895 - - - - - - - - - - - - -

LCL 0.0015 0.0269 0.4922 0.0668 - - - - - - - - - - - -

LN 0.0015 0.0015 0.0015 0.0015 0.0015 - - - - - - - - - - -

LNP 0.0015 0.0028 0.0015 0.0015 0.0015 0.1573 - - - - - - - - - -

LT 0.0015 0.0015 0.0015 0.0015 0.0015 0.0015 0.0015 - - - - - - - - -

OR 0.0015 0.0015 0.0015 0.0015 0.0015 0.0015 0.0015 0.0015 - - - - - - - -

RB 0.0015 0.4431 0.4852 0.8150 0.0747 0.0015 0.0028 0.0015 0.0015 - - - - - - -

RCB 0.0015 0.1396 0.2887 0.6099 0.1288 0.0015 0.0015 0.0015 0.0015 0.7135 - - - - - -

RCL 0.0015 0.0466 0.7546 0.2887 0.7532 0.0015 0.0015 0.0015 0.0015 0.2369 0.2309 - - - - -

RN 0.0015 0.0015 0.0015 0.0015 0.0015 0.9140 0.0611 0.0015 0.0015 0.0015 0.0015 0.0015 - - - -

RNP 0.0015 0.0095 0.0028 0.0042 0.0015 0.0015 0.5429 0.0015 0.0015 0.0207 0.0108 0.0015 0.0028 - - -

RT 0.0015 0.0015 0.0015 0.0015 0.0015 0.0015 0.0015 0.8099 0.0015 0.0015 0.0015 0.0015 0.0015 0.0015 - -

TO 0.0015 0.0015 0.0015 0.0015 0.0015 0.0015 0.0015 0.0015 0.0015 0.0015 0.0015 0.0015 0.0015 0.0015 0.0015 -

TT 0.0015 0.1093 0.0305 0.1860 0.0082 0.0015 0.0145 0.0015 0.0015 0.1948 0.3516 0.0145 0.0015 0.0157 0.0015 0.0015

P value adjustment method: fdr

Legend

LN Left nostril

RN Right nostril

LNP Left nasopharynx

RNP Right nasopharynx

TO Hard palate

BO Floor of mouth

OR Oropharynx

LT Left tonsil

RT Right tonsil

TT Trachea (proximal)

BT Trachea (distal)

LB Left caudal bronchus (primary)

RB Right caudal bronchus (primary)

RCB Right cranial bronchus (primary)

LCL Left caudal bronchi (secondary)

RCL Right caudal bronchi (secondary)

CR Right cranial bronchi (secondary)
